# *Clostridium difficile* Infection–Daily Symptoms (CDI-DaySyms™) questionnaire: psychometric characteristics and responder thresholds

**DOI:** 10.1186/s12955-019-1142-9

**Published:** 2019-05-03

**Authors:** George H. Talbot, Leah Kleinman, Evan Davies, Elke Hunsche, Dennis Revicki, Laurie Roberts, Daniel Rosenberg, Carl Erik Nord

**Affiliations:** 1 Talbot Advisors LLC, PO Box 2121, Anna Maria, FL 34217 USA; 2 Evidera, Bethesda, MD USA; 3 Actelion Pharmaceuticals Ltd., Allschwil, Switzerland; 4 Department of Laboratory Medicine, Karolinska Institutet, Karolinska University Hospital, Stockholm, Sweden; 5 Present Address: Myovant Sciences GmbH, Basel, Switzerland; 6 Present Address: GlaxoSmithKline, Collegeville, Philadelphia, USA

**Keywords:** *Clostridium difficile* infection, *Clostridium difficile*–associated diarrhea, Symptoms, Patient-reported outcomes, Psychometric validation

## Abstract

**Background:**

The purpose of the current study was to determine the final content validation, psychometric characteristics, clinically meaningful improvement, and responder thresholds of the *Clostridium difficile* infection (CDI)–Daily Symptoms (CDI-DaySyms™) patient-reported outcome (PRO) questionnaire.

**Methods:**

This validation study was part of two phase III studies (NCT01987895 and NCT01983683) conducted in patients with mild-to-moderate or severe CDI who completed the CDI-DaySyms™ daily throughout the treatment period. The questionnaire was evaluated in three stages: final PRO item content validation (Stage I); psychometric evaluation of reliability and construct validity (Stage II); and determination of clinically meaningful improvement and responder thresholds using distribution-based methods (Stage III).

**Results:**

The analysis included 168 patients. Most patients were female and Caucasian with mild-to-moderate CDI. The mean age was 57.1 years. Initial item analysis supported by confirmatory factor analysis demonstrated the relevance of 10 items grouped into three distinct domains (Diarrhea Symptoms, Abdominal Symptoms, and Systemic/Other Symptoms). Domain scores demonstrated acceptable internal consistency and test-retest reliability, were sensitive to change, and correlated in expected directions with other relevant symptom and disease-severity measures. Responder thresholds were defined as score changes of − 1.00, − 0.80, and − 0.70 in the Diarrhea Symptoms, Abdominal Symptoms, and Systemic/Other Symptoms domains, respectively.

**Conclusions:**

The CDI-DaySyms™ is a valid measure of patient-reported CDI symptoms, with good measurement properties, which supports its utility as an endpoint in clinical studies. Further studies confirming responder thresholds based on anchor-based methods are required.

**Trial registration:**

NCT01987895, registered November 20, 2013; NCT01983683, registered November 14, 2013.

**Electronic supplementary material:**

The online version of this article (10.1186/s12955-019-1142-9) contains supplementary material, which is available to authorized users.

## Background

*Clostridium difficile* infection (CDI) imposes a major public health burden with increasing incidence, severity, and mortality, along with more than $1 billion of associated medical costs per annual estimates in the United States [1–4]. *C. difficile* infection has been reported in acute care, community settings, and long-term care facilities, with recurrence of CDI following initial treatment being reported in up to 20 to 30% of patients [5, 6]. The clinical manifestations of CDI include diarrhea of varying severity, colitis, systemic toxic shock, and death [1, 2]. Traditionally, CDI severity has been evaluated through clinician assessment of medical history, physical examination results, and methods such as imaging and colonoscopy [7, 8]. However, clinical assessment may not clearly depict the full spectrum of signs and symptoms experienced by the patient. Patient-reported outcomes (PROs) provide the patients’ perspectives on their disease, which are not available from clinical assessments, and have become increasingly critical to the drug approval process [9]. Patient-reported symptoms of CDI include reduced appetite, diarrhea, abdominal pain, loss of control over bowel function, lack of energy, and fatigue [10].

Currently, no PRO questionnaire is available that is specific to the symptoms of CDI, has validated content, and has clearly-defined psychometric characteristics based on the criteria provided in the Food and Drug Administration’s (FDA) PRO Guidance for Industry [11]. Cdiff32, the only disease-specific questionnaire available, is not validated according to FDA standards and it reports on overall health-related quality of life in patients with CDI [12]. Other available PRO instruments/questionnaires such as the Gastrointestinal Symptom Rating Scale (GSRS) [13, 14], the Inflammatory Bowel Disease Questionnaire [15], the IBS-36 measure for irritable bowel syndrome [16], and the Digestive Health Status Instrument [17] are not validated in CDI, do not cover all relevant symptoms of CDI, and are therefore not appropriate for use in CDI.

In response to the lack of an adequate disease-specific daily diary (PRO) covering relevant patient-reported symptoms, the CDI-Daily Symptoms (CDI-DaySyms™) was developed. It measures the broad range of local and systemic CDI symptoms that patients report as meaningful [18].

Before a questionnaire can be used in a clinical trial to support efficacy assessments and potential labelling claims, its content validity, psychometric characteristics, and clinically-meaningful change thresholds must be assessed in the target patient population [11].

The purpose of the current study was to quantitatively support the final content validation and to determine the final scoring algorithm of the questionnaire. The study was conducted in selected sites participating in two phase III efficacy and safety studies of CDI patients. In addition, psychometric characteristics such as reliability (test-retest and internal consistency), construct and known-groups validity, and sensitivity to change of the CDI-DaySyms™ were assessed. Finally, interpretation guidelines were developed for clinically-meaningful improvement ranges and identification of responder thresholds.

## Methods

### Study design

This validation study was conducted in the United States, Canada, Australia, Korea, and Europe between 12 March 2014 and 16 May 2016. The study aimed to include approximately 165 patients based on sample-size calculations for all planned psychometric analyses. Patients with mild-to-moderate or severe CDI were included irrespective of treatment allocation (i.e. all analyses were conducted with blinded data). The study was conducted as a sub-study of the International Multi-center Program Assessing Cadazolid Treatment (IMPACT), which comprised two phase III, double-blind, double-dummy, randomized, parallel-group studies (NCT01987895 and NCT01983683), and was conducted under a separate protocol and statistical analysis plan.

The development of the CDI-DaySyms™ followed the recommendations found in the FDA’s PRO Guidance for Industry [11]. Recent recommendations for establishing clinically-meaningful changes and responder thresholds were also considered [19]. A study Steering Committee of clinical experts provided guidance on the study design, interpretation of results, PRO revision, PRO validation, and determination of responder thresholds.

The study consisted of a screening period of up to 48 h from Days − 2 to 1, followed by random assignment to study drug on Day 1. The treatment period started with the first dose of study drug on Day 1 and lasted until the end of treatment (EOT) on the day of the last dose of study drug, with a follow-up period of up to 4 days. The treatment period included a visit on Day 5 or 6, on site or by telephone (Visit 2), and a visit on Days 8 to 11 (Visit 3). Patient-reported demographic and site-reported clinical information were collected at screening. Patients completed the CDI-DaySyms™ daily up to EOT + 2 to 4 days (Visit 4). Additional questionnaires were administered to describe the population and for use in the psychometric evaluation of the CDI-DaySyms™. Patients completed the Patient Global Assessment of Severity (PGA-S) scale, the GSRS, the Activities of Daily Living (ADL) questionnaire, and the Patient-reported Overall Health Scale at Visits 1 and 3 [13, 14, 20, 21]. Clinicians completed the Clinical Global Impression of Severity (CGI-S) at Visits 1, 3 and 4 and the Clinical Global Impression of Change (CGI-C) scales at Visits 2, 3 and 4. Details related to study visits and assessment schedules are provided in Additional file 1: Table S1.

The research protocol was approved by the Independent Ethics Committee or Institutional Review Board at each participating site. All patients provided written informed consent for one of the IMPACT studies and for this validation sub-study. The CDI-DaySyms™ was translated according to the current best practices [22].

### Patient population

A subset of sites from the IMPACT studies participated in the validation sub-study and were selected based on their willingness to participate and their likelihood of recruiting at least five patients. Planned enrolment was approximately 165 patients. Eligible patients were at least 18 years old, had a diagnosis of mild-to-moderate or severe CDI, with first occurrence or first recurrence within 3 months prior to randomization, and had diarrhea (defined as > 3 unformed bowel movements) within the 24 h prior to randomization along with *C difficile* toxin detected in stool (determined with enzyme immunoassay). A full list of inclusion and exclusion criteria is provided in Additional file 1.

### Statistical analyses for current study

A statistical analysis plan was created a priori for conducting the psychometric evaluation of the draft questionnaire. The evaluation of the questionnaire was performed in three stages: determination of the final PRO item content and scoring algorithm (Stage I), psychometric evaluation of reliability and construct validity (Stage II), and determination of clinically-meaningful improvement and responder thresholds (Stage III).

The Validation Analysis Set comprised patients who had confirmed CDI, had taken study medication for at least 5 days, and had at least one postbaseline assessment. The demographic and baseline disease characteristics were summarized with descriptive statistics.

#### Stage I: final PRO item content validation

The draft CDI-DaySyms™ comprised 13 items with the following response options: none = 0, mild = 1, moderate = 2, severe = 3, and very severe = 4. Items were evaluated for redundancy and poor item performance characteristics indicating potential for removal. Item performance was evaluated using Day 1 data, including assessment of floor/ceiling effects (> 30%), missing data, and strong inter-item correlations (r > 0.80) indicating potential redundancy or low correlations (r < 0.20) indicating a weak relationship with other items.

Exploratory factor analyses (EFA) at Day 1 examined the underlying domain structure among the items using orthogonal and oblique rotations. Eigen values were examined to determine the optimal number of factors [23]. The initial number of factors was specified a priori as three. EFAs were conducted using Mplus software [24].

Rasch analysis was used to examine item properties in relation to the underlying construct being measured. Items with negative fit residual values of less than − 3.0, suggesting an overfitting item, and items with high positive residual values of more than 3.0, suggesting an underfitting item, were flagged for potential deletion. RUMM2030 was used for the Rasch analysis [25].

Differential item functioning examined differences in scores between patients with first occurrence and patients with first recurrence of CDI. Differential item functioning was assessed using the Bonferroni correction for multiple comparisons. Confirmatory factor analysis using Day 2 data was used to confirm the final factor structure, after elimination of items based on item-based analyses, previous qualitative research results, and discussion with the Steering Committee. Data from Day 2 was chosen as, given the efficacy of the CDI treatments, data from Day 5, Day 7, or indeed any other day, would not have been appropriate due to the rapid resolution of symptoms. Hence, selection of Day 2 ensured that patients were experiencing variations in symptoms and represented a different set of data for the confirmatory factor analysis. A scoring algorithm for the CDI-DaySyms™ was developed, including recommendations for handling missing data. The scoring algorithm was developed following confirmatory factor analysis. Further details are provided in the Methods section of Additional file 1.

#### Stage II: psychometric evaluation (measurement characteristics)

Validity and reliability testing was conducted for the final domains identified in Stage I. Test-retest reliability was conducted using intraclass correlation coefficients (ICCs) in the subset of patients who had questionnaire data on Days 9 and 10. Since, we had no measure of patient stability beyond symptom severity (which changed rapidly due to treatment), the decision was made to examine test-retest reliability while patients were still on treatment, but when they were likely clinically stable. Thus, the two-day test-retest period at the end of the on-treatment period was selected. ICCs of at least 0.70 were considered to demonstrate good test-retest reliability for the domain scores. An ICC of 0.4 to 0.7 indicates moderate test-retest reliability, and an ICC of less than 0.4 indicates low test-retest reliability [26, 27].

Internal consistency reliability measured the extent to which the items correlated with the other items within their domain and was assessed using Cronbach’s alpha on Day 1 data. Values greater than 0.70 were considered acceptable [28].

Construct validity includes both concurrent and divergent validity. Construct validity was assessed through evaluation of the correlation between the CDI-DaySyms™ domain scores and relevant GSRS scores, ADL questionnaire scores, and Patient-reported Overall Health Scale scores, as well as PGA-S and CGI-S scale scores at baseline (Visit 1). Construct validity was assessed using Spearman correlation coefficients.

Known-groups validity at baseline was evaluated using analysis of covariance with a fixed effects model that compared initial and change scores between visits on the CDI-DaySyms™ PRO questionnaire between groups that differed on the basis of scores on the Patient Global Assessment of Severity and the Clinical Global Impression of Severity scales. Pairwise comparisons between levels (i.e., severe vs moderate, severe vs mild) were performed using Scheffé’s test adjusting for multiple comparisons and to determine whether domain scores were distinguishable for groups which differed on a key indicator such as severity levels, based on the CGI-S or the PGA-S scales.

For analyses of sensitivity to change from baseline to EOT, improvement was defined as an improvement of 1 point in the CGI-S scale score or at least “minimally better” in the CGI-C scale score. Sensitivity to change was analyzed using a repeated-measures analysis of variance to compare the difference in mean score changes up to each time point in each group. Further details are provided in the Methods section of Additional file 1.

#### Stage III: determination of clinically-meaningful improvement and responder thresholds

Distribution-based methods were used to determine clinically-meaningful change because almost all patients improved by Day 5 or 6, which was the earliest time point when one of the anchor scales was assessed post-baseline. Hence, anchor-based methods were not appropriate for use as the primary method. Clinically-meaningful improvement ranges for CDI-DaySyms™ domain scores were identified by: 1/2 standard deviation (1/2SD), standard error of measurement (SEM), and effect size on Day 3, supported by anchor-based methods at Day 5 or 6. The CGI-C scale was determined to be the best anchor to inform the analyses because it was completed on Day 5 or 6. The responder threshold was the upper boundary of the range for clinically-meaningful improvement.

## Results

### Participant sample

The study enrolled 181 patients, 168 of whom met the criteria for inclusion into the Validation Analysis Set. Patient demographics and baseline disease characteristics are shown in Table 1. The analysis included 168 patients. Most of the patients were female and White, with mild-to-moderate CDI. The mean age was 57.1 years, and most patients had experienced their first occurrence of CDI.

**Table 1:** Summary of patient demographics and baseline disease characteristics

Patient characteristics	Validation analysis set (*N* = 168)
Age (y)	
Mean (SD)	57.1 (16.9)
Median (range)	60 (23.0–92.0)
Sex, n (%)	
Female	114 (67.9)
Race, n (%)	
White	157 (93.5)
Black/African-American	6 (3.6)
Asian	4 (2.4)
Other	1 (0.6)
CDI disease severity, n (%)	
Mild-to-moderate	137 (81.5)
Severe	19 (11.3)
Missing information	12 (7.1)
Status at admission, n (%)	
Inpatient	76 (45.2)
Outpatient	92 (54.8)
CDI episode type (investigator assessment in CRF), n (%)	
First occurrence	135 (80.4)
First recurrence	33 (19.6)

*CDI Clostridium difficile* infection, *CRF* case report form, *SD* standard deviation

At baseline, approximately two-thirds of patients (64.3%) rated themselves as having severe or very severe symptoms on the PGA-S while the clinicians rated 13.7% of patients as having severe or very severe symptoms using the CGI-S. Clinicians are likely to rate patients based on their overall experience of treating patients with a range of CDI severity, while patients are focused on their individual experience and impact, which can be perceived as more severe by them. By EOT, when only the clinicians’ assessments were collected, most clinicians rated symptom severity as none (78.0%), with the remaining being mild (11.9%), moderate (6.0%), or missing (4.2%). Patients rated themselves only at Visit 3, with most reporting none (47.6%) or mild (18.5%) symptoms. These results are detailed in Additional file 1: Table S2.

### Stage I: final PRO item content validation

**Fig. 1:**
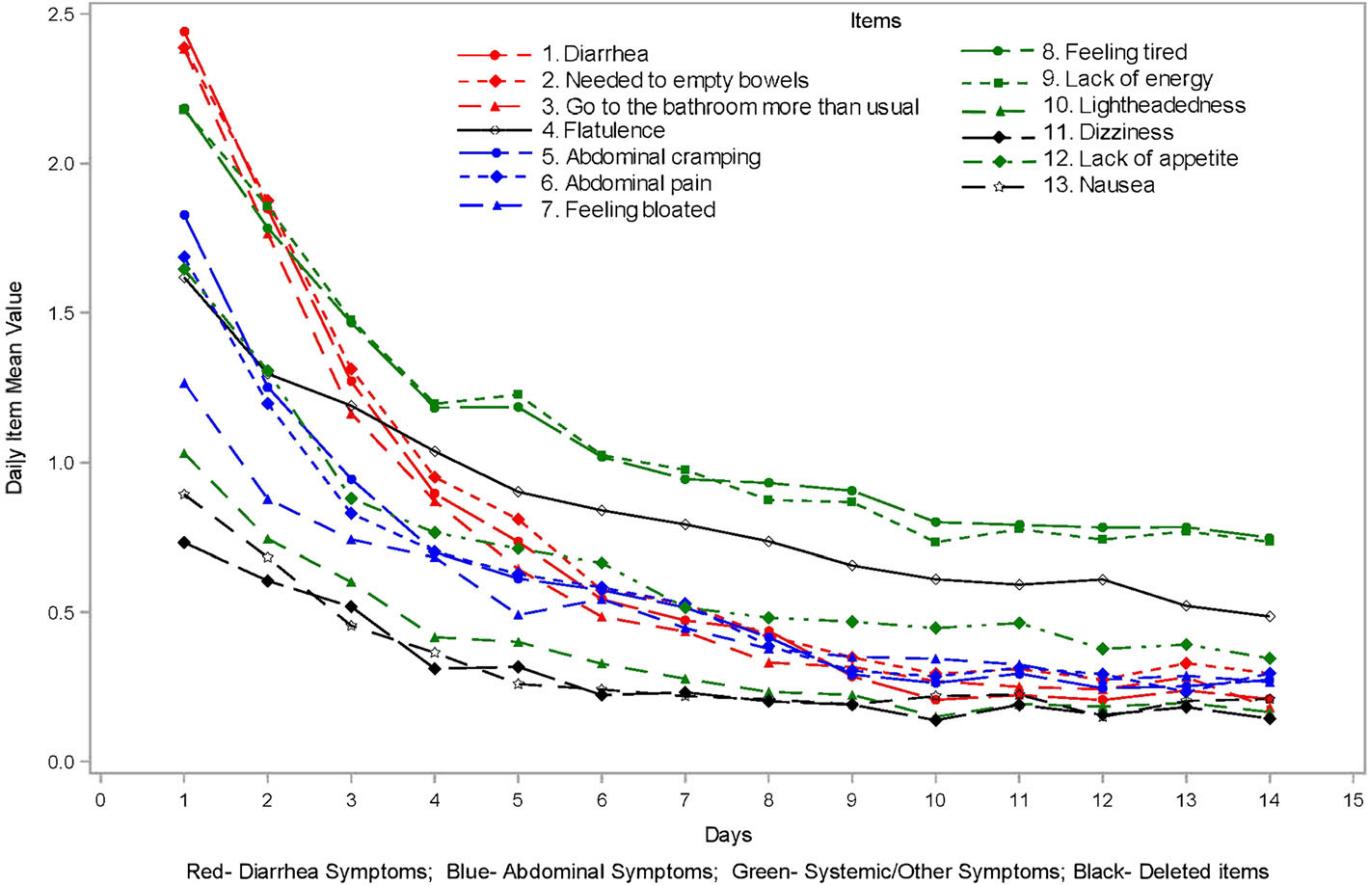
CDI-DaySyms™ PRO questionnaire daily item performance plot (*N* = 168)

At baseline, four items (feeling bloated [38.8%], lightheadedness [44.8%], dizziness [58.5%], and nausea [53.0%]) demonstrated floor effects (> 30%). Inter-item correlations at baseline showed that abdominal cramping and abdominal pain were highly correlated, as were feeling tired and lack of energy. Passing gas had low correlations (all < 0.20) with diarrhea, needing to empty bowels, feeling tired, lack of energy, lightheadedness, dizziness, and lack of appetite. These items were flagged for discussion and possible deletion (Table 2). Individual item score changes over time from baseline to EOT + 4 days are displayed in Fig. 1. Results demonstrated a sharp drop in all symptoms across the entire study.

**Table 2:** CDI-DaySyms™ PRO questionnaire item performance – Day 1

Item	Score, mean (SD) at Day 1	Floor effects at Day 1, %	Ceiling effects at Day 1, %	High correlations > 0.80 with	Low correlations < 0.20 with	Clinical input/Rationale for decision	Item decision
1. Diarrhea	2.4 (1.2)	8.9	16.1	–	Passing gas	Predominant symptom in CDI	Retain
2. Need to empty bowels right away	2.4 (1.2)	8.9	14.3	Bathroom more than usual	Passing gas	Describes intensity of urgency of bowel movements	Retain
3. Need to go to the bathroom more than usual	2.4 (1.2)	11.4	13.8	Need to empty bowels	–	Describes frequency of bowel movements	Retain
4.Passing gas (flatulence)	1.6 (1.1)	17.6	6.1	–	Diarrhea, empty bowels, feeling tired, lack of energy, lightheadedness, dizziness, appetite	Misfit/multidimensionality in Rasch analyses	Delete
5. Abdominal cramping	1.8 (1.3)	22.6	10.7	Abdominal pain	–	According to experts, pathophysiology is different from item 6	Retain
6. Abdominal pain	1.7 (1.3)	22.9	10.8	Abdominal cramping	–	See item 5	Retain
7. Feeling bloated	1.3 (1.3)	38.8	6.7	–	–	Most patients do complain of bloating based on expert input	Retain
8.Feeling tired	2.2 (1.1)	9.5	13.1	Lack of energy	Passing gas	Based on qualitative study, items 8 and 9 are different	Retain
9.Lack of energy	2.2 (1.2)	10.1	13.7	Feeling tired	Passing gas	See item 8	Retain
10. Lightheadedness	1.0 (1.2)	44.8	5.5	–	Passing gas	Experts considered lightheadedness more relevant to CDI than dizziness	Retain
11. Dizziness	0.7 (1.1)	58.5	3.7	–	Passing gas	See item 10	Delete
12. Lack of appetite	1.6 (1.3)	26.9	11.4	–	Passing gas	Experts noted that lack of appetite is relevant in severe disease	Retain
13. Nausea	0.9 (1.2)	53.0	6.0	–	–	The item had a low mean score and high floor effect across patients	Delete

*CDI Clostridium difficile* infection, *CDI-DaySyms™ Clostridium difficile* infection–daily symptoms, *SD* standard deviation

The EFA with the best conceptual fit was multifactorial with three distinct factors. All items had factor loadings greater than 0.30. Rasch analyses were run evaluating the individual domains identified in the three-factor solution. Rasch analysis indicated misfit/multidimensionality for some items. Domain 1 had no misfit/multidimensionality. Domain 2 had some minor misfit/multidimensionality for passing gas and disordered thresholds for abdominal cramping and feeling bloated. Domain 3 had some evidence of misfit for lack of energy and multidimensionality for lack of appetite.

Results from the item evaluation, EFAs, and the Rasch analyses were reviewed with the Steering Committee. Passing gas, dizziness, and nausea were deleted based on the totality of evidence (Table 2). These three items were removed from the CDI-DaySyms based on the statistical analyses and steering committee input as to specificity of the symptom to CDI. “Passing gas” was removed based on misfit and multidimensionality found during the Rasch analyses; low correlations with several of the items indicated that it may not be a relevant component of CDI symptom concepts. Dizziness was removed based on the analyses and the Steering Committee noting that the term dizziness was not relevant to the disease. Nausea was the final item removed with low endorsement across disease severity and a recommendation by the Steering Committee.

**Fig. 2:**
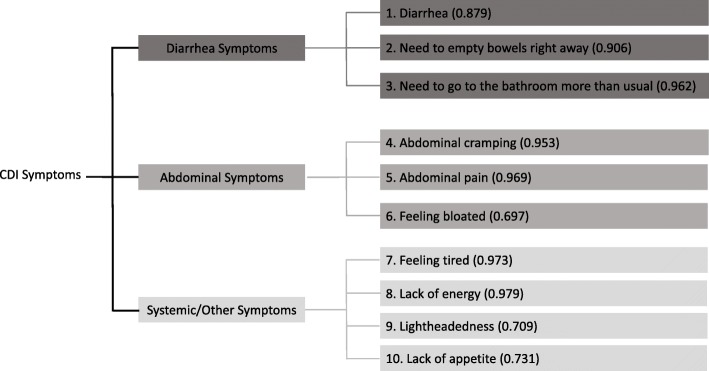
CDI-DaySyms™ PRO questionnaire conceptual framework with confirmatory factor analysis (Day 2)

Two items, abdominal cramping and passing gas, had nonuniform differential item functioning but were nonsignificant after Bonferroni correction. The confirmatory factor analysis on Day 2 showed three factors with confirmatory fit indices of 0.995, indicating excellent model fit. The three domains (containing 10 items) identified are labelled in a conceptual framework (Fig. 2). Factor loadings were as follows: Diarrhea Symptoms domain, 0.879–0.962; Abdominal Symptoms domain, 0.697–0.969; and Systemic/Other Symptoms domain, 0.709–0.979.

The final scoring algorithm was based on the questionnaire’s five-point Likert scale. Likert responses were converted to numerical values as follows: none = 0, mild = 1, moderate = 2, severe = 3, and very severe = 4. Item scores within each domain were averaged to provide three separate domain scores. Based on factor analyses, this questionnaire does not support a total score.

Since diarrhea is the cardinal symptom of CDI, the Steering Committee advised that missing items in that domain be treated as follows: if Item 1 (diarrhea) was available, then a domain score was calculated, even in cases where both Item 2 (need to empty bowels right away) and Item 3 (needing to go to the bathroom more than the usual) were missing. In all other cases, if greater than 50% of the items were missing, then the domain was marked as missing. For the other two domains (Abdominal Symptoms and Systemic/Other Symptoms), if greater than 50% of items were missing, the domain was to be marked as missing.

### Stage II: psychometric evaluation (measurement characteristics)

#### Reliability

Good test-retest reliability was observed for Abdominal and Systemic/Other Symptoms domains (ICC = 0.83), while the Diarrhea Symptoms domain (ICC = 0.62) was below the threshold of 0.70 (Additional file 1: Table S3). All domains demonstrated excellent internal consistency reliability with Cronbach’s alphas for the domains (Diarrhea Symptoms: 0.92; Abdominal Symptoms: 0.82; and Systemic/Other Symptoms: 0.85).

#### Validity

Convergent validity was demonstrated, with mostly moderate correlations between CDI-DaySyms™ domains and other measures that were conceptually equivalent. Conversely, lower correlations were found between CDI-DaySyms™ domains and measures that were conceptually dissimilar, demonstrating divergent validity (Table 3). Overall, results suggest that CDI-DaySyms™ PRO questionnaire measures concepts that are unique to CDI and not fully covered by other questionnaires.

**Table 3:** Construct validity (convergent and divergent validity): correlation between CDI-DaySyms™ PRO questionnaire symptoms and other PROs

	N	CDI-DaySyms™ symptoms^a^ at baseline		
		Diarrhea symptoms	Abdominal symptoms	Systemic/Other symptoms
GSRS				
Abdominal pain	158	0.21*	0.46***	0.39***
Reflux	158	0.27**	0.31***	0.40***
Indigestion	158	0.13	0.39***	0.22*
Diarrhea	158	0.39***	0.27**	0.22*
Constipation	157	0.09	0.16	0.17*
ADL questionnaire–total score	157	0.17*	0.25*	0.38***
Patient-reported overall health scale				
Self-rated health (item 1)	156	−0.12	0.08	−0.13
Overall quality of life (item 2)	155	−0.05	0.07	−0.12
Rating of physical health (item 3)	157	−0.14	0.08	−0.17*
Mental health (item 4)	157	−0.01	0.15	−0.13
Global rating of pain (item 5)	156	0.26*	0.52***	0.31***
Fatigue (item 6)	156	0.27**	0.26*	0.53***
PGA-S scale	159	0.43***	0.36***	0.28**
CGI-S scale	167	0.25**	0.29**	0.22*

*ADL* activities of daily living, *CDI Clostridium difficile* infection, *CDI-DaySyms™ Clostridium difficile* infection–daily symptoms, *CGI-S* clinical global impression of severity, *GSRS* gastrointestinal symptom rating scale, *PGA-S* patient global assessment of severity, *PRO* patient-reported outcome

^a^Pearson product moment correlations or Spearman-rank correlations

*p < 0.05; **p < 0.001; ***p < 0.0001

**Fig. 3:**
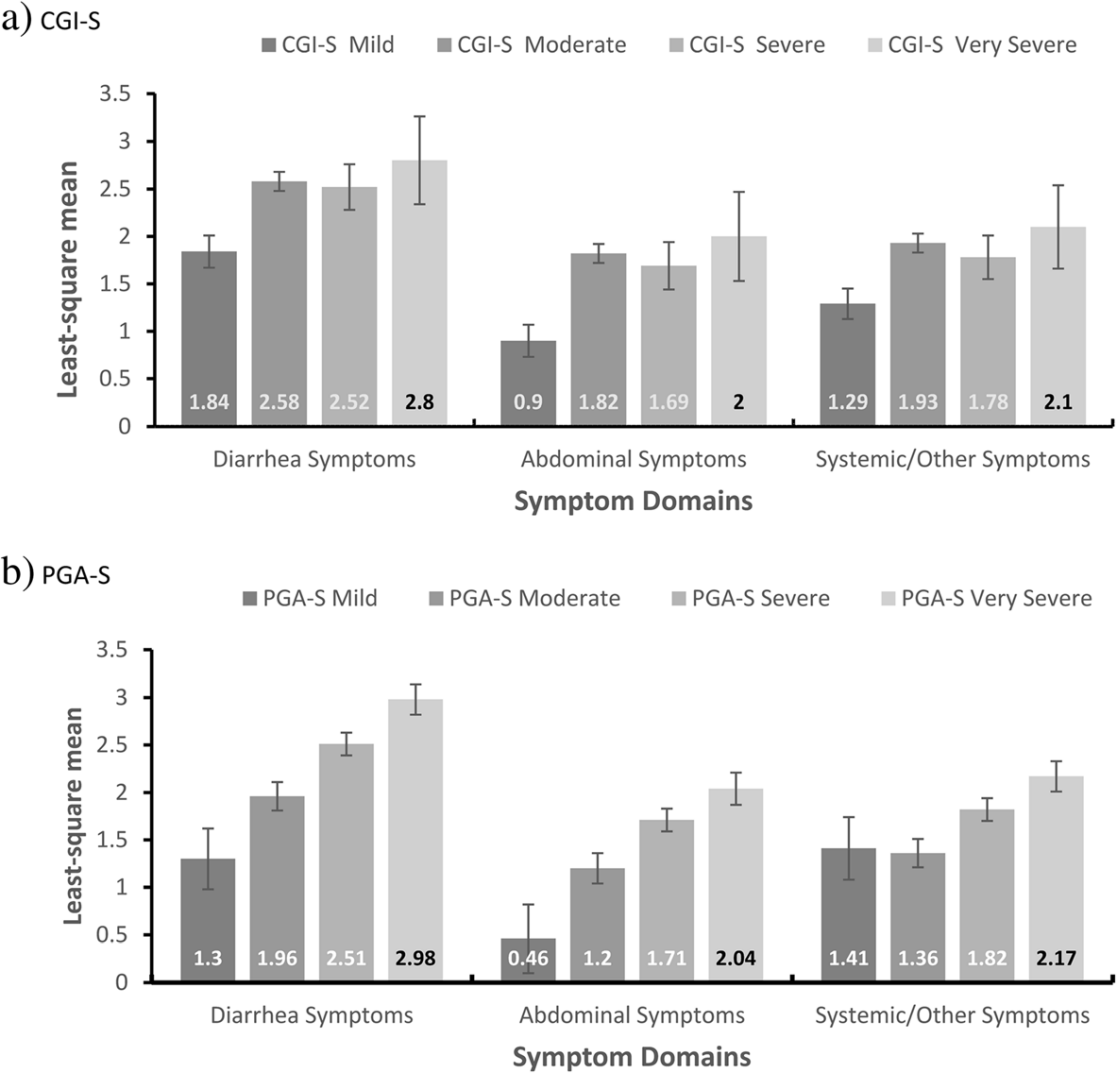
Known-groups validity: ANOVA of CDI-DaySyms™ PRO questionnaire and the CGI-S and PGA-S scales at baseline. Pairwise comparisons between domain LS means were performed using Scheffé’s test adjusting for multiple comparisons. Comparisons were: 1 = Mild vs Moderate; 2 = Mild vs Severe; 3 = Mild vs Very Severe; 4 = Moderate vs Severe; 5 = Moderate vs Very Severe; 6 = Severe vs Very Severe. Significance levels: *p < 0.05; **p < 0.001; ***p < 0.0001. **a** Statistically-significant CGI-S pairwise comparisons were Diarrhea Symptoms for comparison = 1**; Abdominal Symptoms for comparison = 1***; and Systemic/Other Symptoms for comparison = 1**. **b** Statistically-significant PGA-S pairwise comparisons were Diarrhea Symptoms for comparisons = 2**, 3***, 4*, 5***; Abdominal Symptoms for comparisons = 2*, 3**, 5**; and Systemic/Other Symptoms for comparison = 5**. Abbreviations: ANOVA, Analysis of Variance; CDI-DaySyms™, *Clostridium difficile* Infection–Daily Symptoms; CGI-S, Clinical Global Impression of Severity; LS, least-square; PGA-S, Patient Global Assessment of Severity; PRO, patient-reported outcome

At baseline, all three domains demonstrated known-groups validity among the categories of mild, moderate, severe, and very severe based on the severity categories of the CGI-S (*p* = 0.008 to *p* = 0.0001) and PGA-S (*p* = 0.0029 to *p* < 0.0001) scales, respectively. The scores increased as expected from mild to moderate and mild to very severe on both the CGI-S and PGA-S scales. However, due to the small sample size in the more severe categories, there were no increases between the moderate and severe categories on the CGI-S scale (Fig. 3a), whereas the mean score decreased slightly for the Systemic/Other Symptoms domain from mild to moderate on the PGA-S scale (Fig. 3b).

Pairwise comparisons between mild and moderate were statistically significant among CGI-S scale categories (*p* < 0.001). Almost all pairwise comparisons among PGA-S scale categories were significant for the Diarrhea Symptoms and Abdominal Symptoms domains (*p* < 0.05) (Additional file 1: Table S4).

Sensitivity to change using the CGI-C scale was demonstrated at EOT for the Diarrhea Symptoms domain (*p* = 0.0184), whereas the Abdominal Symptoms and Systemic/Other Symptoms domains were nonsignificant at *p* = 0.0807 and *p* = 0.7510, respectively. Sensitivity to change using the CGI-S scale was demonstrated at EOT on the Diarrhea Symptoms (*p* = 0.0003) and the Systemic/Other Symptoms domains (*p* = 0.0045), whereas the Abdominal Symptoms domain demonstrated a *p* value of *p* = 0.0595.

### Stage III: determination of clinically-meaningful improvement and responder thresholds

The distribution measure 1/2 SD was 0.54, the SEM was 0.92, and the effect size was − 1.08 for the Diarrhea Symptoms domain. For the Abdominal Symptoms domain, the 1/2 SD was 0.55, the SEM was 0.78, and the effect size was − 0.69, whereas for the Systemic/Other Symptoms domain, the 1/2 SD was 0.51, the SEM was 0.66, and the effect size was − 0.63. Results of the distribution-based analyses, supported by anchor-based methods, were triangulated to estimate a clinically-meaningful improvement range for each domain (Diarrhea Symptoms, − 1.00 to − 0.55; Abdominal Symptoms, − 0.80 to − 0.55; and Systemic/Other Symptoms, − 0.70 to − 0.50; Additional file 1: Figure S1).

Responder thresholds were defined as score changes of − 1.00, − 0.80, and − 0.70 for the Diarrhea, Abdominal Symptoms, and Systemic/Other Symptoms domains, respectively. Responder thresholds were met by over half of the patients for all domains (Diarrhea Symptoms: 67.5%; Abdominal Symptoms: 59.6%; Systemic/Other Symptoms: 56.0%; Additional file 1: Table S5).

## Discussion

The CDI-DaySyms™ is the first daily symptom diary measuring the broad range of CDI symptoms, which patients in a prior study reported as relevant and meaningful [18]. The development and validation of the questionnaire were rigorously completed in accordance with the FDA’s PRO Guidance for Industry [11]. This paper presents the quantitative evidence to support the final content validation, psychometric characteristics, and clinically-meaningful improvement ranges and responder thresholds for the CDI-DaySyms™.

The Cdiff32 is the only other available disease-specific questionnaire for measuring symptoms of CDI. However, it differs from the CDI-DaySyms™ in that the domains focus on more distal concepts, such as the burden and/or impact of CDI symptoms on patients’ lives, including daily activities, diet, and sleep [12]. Additionally, the Cdiff32 has a week-long recall period, which would not be appropriate for CDI symptoms, given the rapidity with which the disease can respond to therapy. Thus, the CDI-DaySyms™ represents an important and useful new symptom-based endpoint for application in clinical trials evaluating treatments for CDI as well as clinical practice.

In the current study, most patients had disease of mild-to-moderate severity and were experiencing their first occurrence of CDI; they were generally representative of the CDI population encountered in routine clinical practice and expected to be recruited in future clinical trials. Epidemiologic surveys also support this conclusion about the generalizability of the study population [29, 30].

Assessment of test-retest reliability in an acute disease in a clinical trial setting is difficult because of the rapid symptom improvement. The expectation in the case of CDI was that symptoms would begin to demonstrate improvement 24 to 48 h following treatment, making it difficult to examine test-retest reliability early in the clinical trial. To overcome this challenge, these analyses were conducted during Days 9 to 10, when symptom change had slowed down. This approach demonstrated acceptable ICCs for the Abdominal Symptoms and Systemic/Other Symptoms domain (ICC = 0.83), and an ICC that was slightly below the recommended threshold for the Diarrhea Symptoms domain (0.62), which might reflect the ongoing daily variability in diarrhea. The authors believe that test-retest reliability of the CDI-DaySyms™ has been sufficiently established, although it would be interesting to examine this in a more stable patient population.

It is important to establish responder thresholds, i.e., the amount of change on a PRO measure that is meaningful from the perspective of patients, both for application in clinical trials and clinical practice. Anchor-based methods could not be used as the primary method to determine meaningful change thresholds, because most of the patients had improved by Days 8 to 11 (when the PGA-S and CGI-S anchor measures were administered). Hence, distribution-based methods were used for the main analyses for determining meaningful change and responder thresholds, with anchor based methods being used for triangulation and to inform upper bounds of the range. Day 3 data were selected based on discussions with clinical experts, as data collection at this time represented the most opportune time to examine meaningful change, because response to therapy is generally assessed after 2 to 3 days of treatment in clinical practice. Clinically-meaningful improvement and responder definitions were identified, with responder thresholds being met by over half of the patients for all domains. Further studies to confirm the responder thresholds using anchor-based methods are required.

## Conclusions

The CDI-DaySyms™ is a valid measure of diarrhea and other CDI-related symptoms. It assesses patient-relevant CDI symptoms and can be used to monitor CDI patient improvement and response to treatment. The CDI-DaySyms™ showed good measurement properties and these strong psychometric characteristics support its utility as an endpoint in clinical studies. In addition, as the first PRO questionnaire to capture a complete range of symptoms associated with CDI, it could be a useful tool for healthcare practitioners in a clinical practice setting.

## Additional file

Additional file 1: **Table S1.** Study visit and assessment schedule. **Table S2.** CGI-S and PGA-S descriptive statistics (*N* = 168). **Table S3.** Test-retest reliability: Day 9 compared with Day 10. **Table S4.** Known-groups validity: ANOVA of CDI-DaySyms™ PRO questionnaire and the CGI-S and PGA-S scales at baseline. **Table S5.** Proportion of patients who met meaningful change thresholds (*N* = 166). **Figure S1.** Meaningful change triangulation: Day 3 and Days 5/6 (Visit 2). (DOCX 1878 kb)

## Abbreviations

ADL: Activities of daily living
CDI: *Clostridium difficile* infection
CDI-Daily symptoms: CDI-DaySyms™
CGI-C: Clinical global impression of change
CGI-S: Clinical global impression of severity
EFA: Exploratory factor analyses
EOT: End of treatment
FDA: Food and Drug Administration
GSRS: Gastrointestinal symptom rating scale
ICCs: Intraclass correlations
IMPACT: International multi-center program assessing cadazolid treatment
PGA-S: Patient global assessment of severity
PROs: Patient-reported outcomes
SEM: Standard error of measurement
