# Supplementary material for: Clostridium difficile Infection–Daily Symptoms (CDI-DaySyms™) questionnaire: psychometric characteristics and responder thresholds
Source: Health Qual Life Outcomes. 2019 May 3;17:77. doi: 10.1186/s12955-019-1142-9 (PMC6499966; doi:10.1186/s12955-019-1142-9)
Supplement: Supplementary file 1 — Table S1. Study visit and assessment schedule. Table S2. CGI-S and PGA-S descriptive statistics (N = 168). Table S3. Test-retest reliability: Day 9 compared with Day 10. Table S4. Known-groups validity: ANOVA of CDI-DaySyms™ PRO questionnaire and the CGI-S and PGA-S scales at baseline. Table S5. Proportion of patients who met meaningful change thresholds (N = 166). Figure S1. Meaningful change triangulation: Day 3 and Days 5/6 (Visit 2). (DOCX 1878 kb) [file 12955_2019_1142_MOESM1_ESM.docx]

**Additional File 1**

**The *Clostridium Difficile* infection–Daily Symptoms (CDI‑DaySyms™) Questionnaire: Psychometric Characteristics and Responder Thresholds**

**Table of Contents**

[Methods 2](#_Toc520887506)

[Phase III Patient Inclusion and Exclusion Criteria 2](#_Toc520887507)

[Statistical analyses for study 4](#_Toc520887508)

[Stage I: Item evaluation and scoring 4](#_Toc520887509)

[Stage II: Psychometric evaluation 5](#_Toc520887510)

[RESULTS 6](#_Toc520887511)

[Supplementary Table 1 Study visit and assessment schedule 6](#_Toc520887512)

[Supplementary Table 2 CGI-S and PGA-S descriptive statistics (N = 168) 8](#_Toc520887513)

[Supplementary Table 3 Test-retest reliability: Day 9 compared with Day 10 9](#_Toc520887514)

[Supplementary Table 4 Known-groups validity: ANOVA of CDI-DaySyms™ PRO questionnaire and the CGI-S and PGA‑S scales at baseline 10](#_Toc520887515)

[Supplementary Table 5 Proportion of patients who met meaningful change thresholds (N = 166) 12](#_Toc520887516)

[Supplementary Figure 1 Meaningful change triangulation: Day 3 and Days 5/6 (Visit 2) 13](#_Toc520887517)

# **Methods**

## Phase III Patient Inclusion and Exclusion Criteria

*Inclusion criteria*

Eligible patients were required to have fulfilled the following inclusion criteria:

1. Signed informed consent prior to any study-mandated procedure.

2. Been male or female^[[1]](#footnote-1),^^[[2]](#footnote-2)^ of at least 18 years of age at the screening visit.

3. Had a diagnosis of mild-to-moderate or severe *Clostridium difficile* infection (CDI; first occurrence or first recurrence within 3 months of randomization) with:

- Diarrhea, defined as a change in bowel habits with more than 3 unformed bowel movements in the 24 hours prior to randomization, and
- Positive *C difficile* glutamate dehydrogenase and toxin A and/or B stool test results, on the same sample collected no more than 72 hours before randomization using an enzyme immunoassay (EIA) test approved by the sponsor (*C. Diff* Quik Chek Complete^®^; additional EIAs were approved by the sponsor if used as standard of care. A full list is provided in the table that follows.)

| ***C difficile* GDH antigen EIA test name** | **Manufacturer** |
| --- | --- |
| *C. Diff* QUIK CHEK Complete^®^ | TechLab |
| *C. Diff* CHEK™-60 | TechLab |
| BD Culturette™ CDT™ | Becton Dickinson |
| Immuno*Card*^®^ *C. difficile* GDH | Meridian Bioscience, Inc. |
| VIDAS^®^ *C. difficile* GDH | Biomérieux |
| Premier^®^ *C. difficile* GDH | Meridian Bioscience, Inc. |

*EIA* enzyme immunoassay, *GDH* glutamate dehydrogenase.

*Exclusion criteria*

Eligible patients were required to have had none of the following exclusion criteria:

1. More than one previous episode of CDI in the 3-month period prior to randomization.
2. Fulminant or life-threatening CDI. If, in the judgment of the investigator, there was a suspicion of fulminant or life-threatening CDI, the presence of any of the following criteria during the 72-hour period prior to randomization and related to the fulminant or life-threatening CDI episode excluded the potential patient from the study:

- Septic shock—systolic blood pressure less than 90 mmHg or a mean arterial pressure less than 70 mmHg in the absence of other causes of hypotension and that persisted despite adequate fluid resuscitation
- Peritonitis
- Ileus
- Toxic megacolon
- Significant dehydration according to investigator judgment
- White blood cell count greater than 30.0 × 10^9^/L
- Core body temperature greater than 40°C

1. Concurrent immediately life-threatening disease or condition (likelihood of death within 72 hours).
2. History of inflammatory colitis (eg, ulcerative colitis or Crohn’s disease, microscopic colitis, collagenous colitis) or chronic abdominal pain or chronic diarrhea of any etiology, or known positive diagnostic test results for enteropathogens.
3. Vomiting or other condition that interfered with the ability to take oral medication or patients with feeding tubes (i.e., when study treatment would have to be given by the feeding tube).
4. Antimicrobial treatment active against CDI administered for more than 24 hours, except metronidazole treatment failures.

## Statistical analyses for study

### Stage I: Item evaluation and scoring

Factor loading of at least 0.3 was the criterion for accepting a factor solution. An acceptable factor solution explained at least 50% of variance and did not have an item loading on more than one factor. Items that did not fit the factor solution were flagged for possible deletion.

Rasch analysis examined whether each item exhibited ordinal scaling with properties: 1) patient who was experiencing a severe/less severe symptom chose severe/less severe response options, respectively; 2) each item measured a range of CDI symptoms and, overall, they covered a wide range of the underlying concepts being measured; and 3) all items formed a unidimensional construct for a domain to be reliable.

Model fit was assessed with the confirmatory fit index and the root mean squared error of approximation. The confirmatory factor analysis model was considered to have good fit if the confirmatory fit index was greater than 0.90 and the root mean squared error of approximation was less than 0.07.

### Stage II: Psychometric evaluation

Concurrent validity involved demonstrating that different measures of the same concept substantially correlated. Divergent validity involved demonstrating that measures with different concepts have lower correlations. Construct validity was assessed using Spearman correlation coefficients, with the assumption that correlations between 0.10 and less than 0.30 implied a weak association (divergent validity); correlations between 0.30 and 0.50 implied a moderate association; and correlations greater than 0.50 implied a strong association (convergent validity).

# **RESULTS**

## Additional file 1: Table S1. Study visit and assessment schedule

| Periods | Name | Screening | Treatment | | | Follow-up |
| --- | --- | --- | --- | --- | --- | --- |
|  | Duration | Up to 48 hours | 10 to 11 days | | | Up to 4 days |
| Visit | Number | 1 | | 2 | 3 | 4 |
|  | Name | Screening | Randomization |  |  |  |
| Evaluation | Time | Day −2 to up to Day 1 (within 48 h) | Day 1 | Day 5 or Day 6  On site or by telephone | Day 8 to Day 11, including premature discontinuation | EOT + 2 to 4 days  On site or by telephone |
| Patient-completed | | | | | | |
| Written informed consent | | X | - | - | - | - |
| GSRS | | X | - | - | X | - |
| ADL questionnaire | | X | - | - | X | - |
| PGA-S scale | | X | - | - | X | - |
| Patient-Reported Overall Health Scale | | X | - | - | X | - |
| CDI-DaySyms™ PRO questionnaire^a^ | |  | | | | |
| Clinician-completed | | | | | | |
| CGI‑S scale | | X | - | - | X | X |
| CGI‑C scale | | - | - | X | X | X |

*ADL* activities of daily living, *CDI-DaySyms™* *Clostridium difficile* infection–daily symptoms, *CGI‑C* clinical global impression of change, *CGI-S* clinical global impression of severity, *EOT* end of treatment, *GSRS* gastrointestinal symptom rating scale, *PGA-S* patient global assessment of severity, *PRO* patient‑reported outcomes

^a^These data were completed daily in the evening as per the protocol of the IMPACT-1 and IMPACT-2 studies

## Additional file 1: Table S2. CGI-S and PGA-S descriptive statistics (N = 168)

|  | CGI-S scale, *n* (%) | PGA-S scale, *n* (%) |
| --- | --- | --- |
| Baseline (Visit 1) |  |  |
| None | 0 (0.0) | 0 (0.0) |
| Very mild | 0 (0.0) | 0 (0.0) |
| Mild | 38 (22.6) | 9 (5.4) |
| Moderate | 106 (63.1) | 42 (25.0) |
| Severe | 18 (10.7) | 70 (41.7) |
| Very severe | 5 (3.0) | 38 (22.6) |
| Missing | 1 (0.6) | 9 (5.4) |
| Visit 3 |  |  |
| None | 103 (61.3) | 80 (47.6) |
| Very mild | 0 (0.0) | 0 (0.0) |
| Mild | 48 (28.6) | 31 (18.5) |
| Moderate | 11 (6.5) | 20 (11.9) |
| Severe | 3 (1.8) | 13 (7.7) |
| Very severe | 0 (0.0) | 7 (4.2) |
| Missing | 3 (1.8) | 17 (10.1) |
| EOT (Visit 4) |  |  |
| None | 131 (78.0) | - |
| Very mild | 0 (0.0) | - |
| Mild | 20 (11.9) | - |
| Moderate | 10 (6.0) | - |
| Severe | 0 (0.0) | - |
| Very severe | 0 (0.0) | - |
| Missing | 7 (4.2) | - |

*CGI-S* clinical global impression of severity, *EOT* end of treatment, *PGA-S* patient global assessment of severity

## Additional file 1: Table S3. Test-retest reliability: Day 9 compared with Day 10

| CDI DaySyms^™^ | N | Score at Day 9 Mean (SD) | Score at Day 10 Mean (SD) | Score Difference Mean (SD)^a^ | T‑value | *P*‑value | Pearson r | ICC^b^ |
| --- | --- | --- | --- | --- | --- | --- | --- | --- |
| Diarrhea symptoms | 157 | 0.3 (0.6) | 0.3 (0.5) | 0.1 (0.5) | 1.64 | 0.1036 | 0.63 | 0.62 |
| Abdominal symptoms | 156 | 0.3 (0.6) | 0.3 (0.6) | 0.0 (0.3) | 0.35 | 0.7235 | 0.76 | 0.83 |
| Systemic/Other symptoms | 157 | 0.6 (0.6) | 0.5 (0.6) | 0.1 (0.4) | 2.66 | 0.0087 | 0.87 | 0.83 |

*CDI-DaySyms™* *Clostridium difficile* infection–daily symptoms, *ICC* intraclass correlation coefficient, *SD* standard deviation

^a^Mean difference = Day 9 – Day 10

^b^ICCs ≥0.70 are considered to demonstrate good test-retest reliability

## Additional file 1: Table S4. Known-groups validity: ANOVA of CDI-DaySyms™ PRO questionnaire and the CGI-S and PGA‑S scales at baseline

|  | CGI-S score of mild | | CGI-S score of moderate | | CGI-S score of severe | | CGI-S score of very severe | | Overall F-test^b^ | | Pairwise comparison (*P*‑value)^c^ |
| --- | --- | --- | --- | --- | --- | --- | --- | --- | --- | --- | --- |
|  | N | LS mean (SE) | N | LS mean (SE) | N | LS mean (SE) | N | LS mean (SE) | F | *P*-value |  |
| CDI-DaySyms™ | | | | | | | | | | | |
| Diarrhea symptoms | 38 | 1.84 (0.17) | 106 | 2.58 (0.10) | 18 | 2.52 (0.24) | 5 | 2.80 (0.46) | 5.10 | 0.0021 | 1** |
| Abdominal symptoms | 37 | 0.90 (0.17) | 105 | 1.82 (0.10) | 18 | 1.69 (0.25) | 5 | 2.00 (0.47) | 7.31 | 0.0001 | 1*** |
| Systemic/Other symptoms | 38 | 1.29 (0.16) | 106 | 1.93 (0.10) | 18 | 1.78 (0.23) | 5 | 2.10 (0.44) | 4.10 | 0.0078 | 1** |
|  | PGA-S score of mild | | PGA-S score of moderate | | PGA-S score of severe | | PGA-S score of very severe | | Overall F-test^b^ | | Pairwise comparison (*P*‑value)^c^ |
|  | N | LS mean (SE) | N | LS mean (SE) | N | LS mean (SE) | N | LS mean (SE) | F | *P*-value |  |
| CDI-DaySyms™ | | | | | | | | | | | |
| Diarrhea symptoms | 9 | 1.30 (0.32) | 42 | 1.96 (0.15) | 70 | 2.51 (0.12) | 38 | 2.98 (0.16) | 11.58 | <0.0001 | 2**, 3***, 4*, 5*** |
| Abdominal symptoms | 8 | 0.46 (0.36) | 42 | 1.20 (0.16) | 69 | 1.71 (0.12) | 38 | 2.04 (0.17) | 8.09 | <0.0001 | 2*, 3**, 5** |
| Systemic/Other symptoms | 9 | 1.41 (0.33) | 42 | 1.36 (0.15) | 70 | 1.82 (0.12) | 38 | 2.17 (0.16) | 4.86 | 0.0029 | 5** |

*ANOVA* analysis of variance, *CDI-DaySyms™* *Clostridium difficile* infection–daily symptoms, *CGI-S* clinical global impression of severity, *GSRS*, gastrointestinal symptom rating scale, *LS* least squares, *PGA-S* patient global assessment of severity, *PRO* patient-reported outcome, *SE*, standard error

^a^Using CGI-S and PGA-S scores from Visit 1

^b^One-way analysis of variance using Scheffé’s post hoc comparisons

^c^Pairwise comparisons between LS means were performed using Scheffé’s test adjusting for multiple comparisons

1: Mild vs Moderate; 2: Mild vs Severe; 3: Mild vs Very Severe; 4: Moderate vs Severe; 5: Moderate vs Very Severe; 6: Severe vs Very Severe

**p* < 0.05; ***p* < 0.001; ****p* < 0.0001

## Additional file 1: Table S5. Proportion of patients who met meaningful change thresholds (N = 166)

| Meaningful change thresholds | Diarrhea symptoms  (−0.55 to −1.0) | | Abdominal symptoms  (−0.55 to −0.80) | | Systemic/Other symptoms  (−0.50 to −0.70) | |
| --- | --- | --- | --- | --- | --- | --- |
|  | Change from baseline | | | | | |
|  | ≤ −1.00 | ≤ −0.55 | ≤ −0.80 | ≤ −0.55 | ≤ −0.70 | ≤ −0.50 |
| Proportion of patients who met meaningful change thresholds (Day 1–Day 3) | 58.4% | 67.5% | 38.6% | 59.6% | 7.0% | 56.0% |

## Additional file 1: Figure S1. Meaningful change triangulation: Day 3 and Days 5/6 (Visit 2)


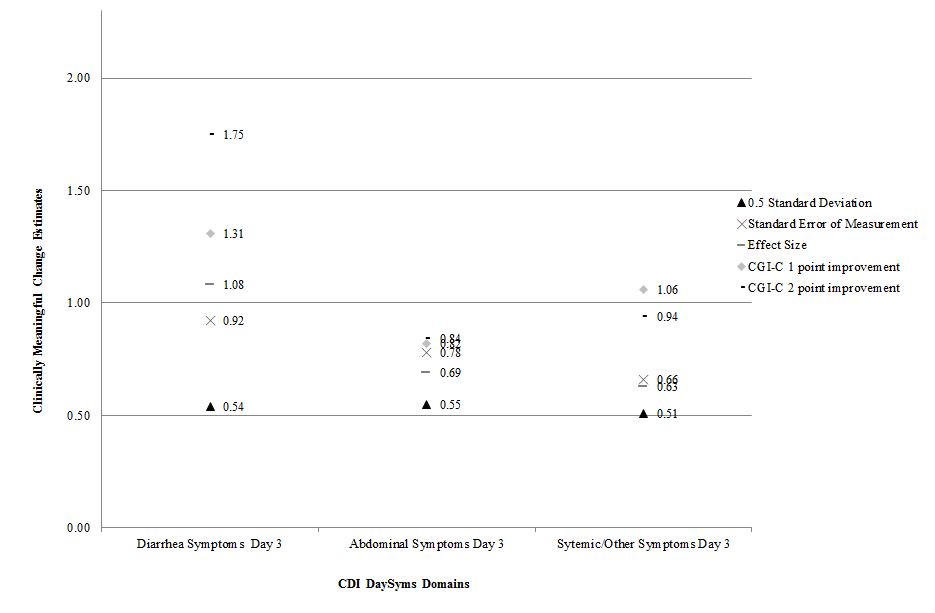


1. Non-pregnant women of childbearing potential:

   A woman was considered to be of childbearing potential unless she met at least one of the following criteria:

   Previous bilateral salpingo-oophorectomy or hysterectomy

   Premature ovarian failure confirmed by a healthcare professional

   XY genotype, Turner syndrome, uterine agenesis

   Postmenopausal, defined as 12 consecutive months with no menses without an alternative medical cause (ICH M3 definition) [↑](#footnote-ref-1)
2. A non-pregnant woman of childbearing potential was eligible only if:

   The absence of pregnancy was confirmed by a negative urine (or plasma/serum) pregnancy test at Visit 1

   She agreed to use one of the following methods of contraception from Visit 1 until 7 days after discontinuation of study treatment: condoms, diaphragm, or contraceptive sponge if used in combination with a spermicide; intra-uterine device; injectable contraceptive agent; levonorgestrel implant; or transdermal contraceptive hormone patch. If a hormonal contraceptive was chosen, it was required to be taken for at least 1 month prior to randomization. Alternatively, a sterilization method (tubal ligation or partner’s vasectomy) was considered acceptable, or she was in a situation of abstinence from intercourse with a male partner, when this was in line with the preferred lifestyle of the patient (e.g., homosexual women or women in a religious order, e.g., nuns)

   In case of oral contraception, an additional method was to be employed, as diarrhea could affect the effectiveness of the oral contraceptive pill. Rhythm methods or the use of a condom by a male partner alone were not considered to be acceptable methods of contraception [↑](#footnote-ref-2)
